# A three-protein signature and clinical outcome in esophageal squamous cell carcinoma

**DOI:** 10.18632/oncotarget.3102

**Published:** 2014-12-31

**Authors:** Hui-Hui Cao, Shi-Yi Zhang, Jin-Hui Shen, Zhi-Yong Wu, Jian-Yi Wu, Shao-Hong Wang, En-Min Li, Li-Yan Xu

**Affiliations:** ^1^ The Key Laboratory of Molecular Biology for High Cancer Incidence Coastal Chaoshan Area, Shantou University Medical College, Shantou, Guangdong, P.R. China; ^2^ Institute of Oncologic Pathology, Shantou University Medical College, Shantou, Guangdong, P.R. China; ^3^ Departments of Oncology Surgery, Shantou Central Hospital, Affiliated Shantou Hospital of Sun Yat-sen University, Shantou, Guangdong, P.R. China; ^4^ Departments of Pathology, Shantou Central Hospital, Affiliated Shantou Hospital of Sun Yat-sen University, Shantou, Guangdong, P.R. China; ^5^ Department of Biochemistry and Molecular Biology, Shantou University Medical College, Shantou, Guangdong, P.R. China; ^6^ Departments of Pathology, Zhuhai People's Hospital, Zhuhai, Guangdong, P.R. China

**Keywords:** ESCC, Kindlin-2, Myosin-9, three-protein signature, prognosis

## Abstract

Current staging is inadequate to precisely predict clinical outcome of esophageal squamous cell carcinoma (ESCC) and determine treatment choices, which vary from operation alone to intensive multimodal regimens. The purpose of this study is to investigate the prognostic values of an immunohistochemistry-based three-protein signature model in patients with ESCC. We determined the protein expression of Annexin II, cofilin 1, ezrin, fascin, kindlin-2, moesin, MTSS1, myosin-9, profilin-1, Rac1, radixin, ROCK2, talin, tensin and villin 1 in a test cohort including 110 formalin-fixed, paraffin-embedded esophageal curative resection specimens by tissue microarrays (TMAs). A three-protein signature elicited from the protein cluster, Annexin II, kindlin-2, and myosin-9, was validated by TMAs on an independent cohort of 147 specimens. The expression of three-protein signature was highly predictive of ESCC overall survival (OS) and disease-free survival (DFS) in both generation and validation datasets. Regression analysis shows that this three-protein signature is an independent predictor for OS and DFS. Furthermore, the predictive ability of these 3 biomarkers in combination is more robust than that of each individual biomarker. This study demonstrates a clinically applicable prognostic model that accurately predicts ESCC patient survival and/or tumor recurrence, and thus could serve as a complement to current risk stratification approaches.

## INTRODUCTION

Esophageal squamous cell carcinoma (ESCC) still remains the most common cancer-induced mortality in China, in particular in areas nearby the Taihang Mountain range and Coastal Chaoshan [1]. Since treatment choices vary from operation alone to intensive multimodal regimens, staging is critical for determination of therapeutic modality, but current markers remain inadequate to precisely predict clinical outcome of ESCC. Therefore, identifying patients at high risk and improving the overall prognosis of the disease are urgent needs for the current clinical management of ESCC [2].

Gene-expression profiling is useful for identifying sets of genes of prognostic importance in various types of cancer [3-6]. Nevertheless, the use of microarrays in clinical practice is limited by the overwhelming number of genes identified by gene profiling, lack of both reproducibility and independent validation, and need for complicated statistical analyses [7]. Different prognostic gene sets are identified when the microarray platform and the analytic strategy vary [8]. Moreover, suitable specimens from patients are still a technical challenge for gene-expression profiling, which requires frozen tissue for analysis [9]. To put these expression profiles into clinical practice, it is essential to identify the appropriate number of genes and develop a profile that can be operated by conventional assay. The aim of this study was to evaluate the expression of 15 cytoskeleton proteins and their correlation with the clinical outcome of ESCC patients. We also develop a technically simple immunohistochemistry-based three-protein signature model for current clinical risk stratification approaches and test its performance using an independent validation dataset of ESCC tissue samples.

## RESULTS

### Immunohistochemical Characteristics of 15 Biomarkers

**Figure 1:**
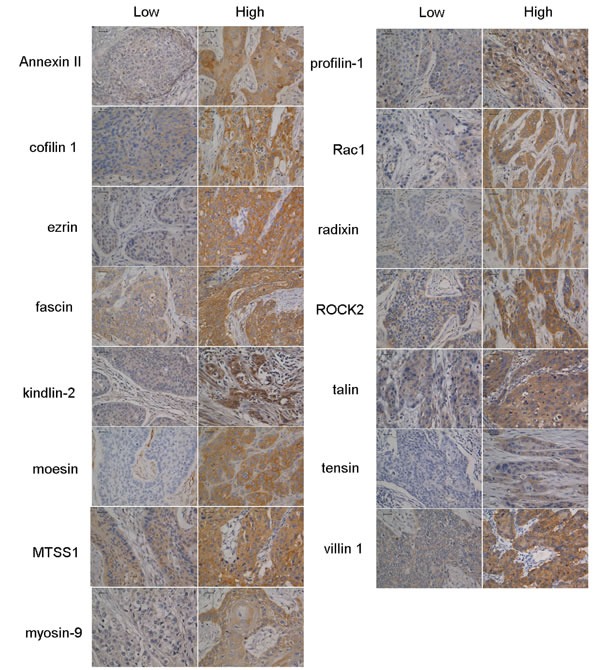
Representative positive expressions of Annexin II, cofilin 1, ezrin, fascin, kindlin-2, moesin, Metastasis suppressor 1 (MTSS1), myosin-9, profilin-1, Ras-related C3 botulinum toxin substrate 1 (Rac1), radixin, Rho-associated coiled-coil containing protein kinase 2 (ROCK2), talin, tensin and villin 1 by immunohistochemistry in tissue microarrays Scale bars = 50 μm.

Initially, more than 15 biomarkers were examined by immunohistochemical staining. Some molecular hallmarks, however, were not optimized successfully or showed ambiguous or no immunostaining. All 15 biomarkers mainly stained the tumour cell cytoplasm and showed a variety of staining patterns of different staining intensity and positive cell percentage. Based on the staining intensity, all biomarkers displayed the four immunostaining phenotypes: negative staining, weakly positive staining, moderate positive staining and strongly positive staining in ESCC. The staining patterns of the 15 biomarkers were focal, scattered and diffuse with different staining intensities. Cofilin 1, ezrin, fascin, moesin, myosin-9, radixin, ROCK2, talin, tensin and villin 1 were constitutively observed in the cytoplasm, and Annexin II was observed in the membrane and cytoplasm. Kindlin-2, MTSS1, profilin-1 and Rac1 not only showed positive cytoplasmic immunostaining, but also strong nuclear immunostaining. The staining pattern of the 15 biomarkers is summarized in Table 1. Representative pictures of the 15 biomarkers with low- and high-expression are shown in Figure 1

**Table 1:** Primary antibodies used in this study

Antibody	Manufacturer	Catalog NO.	Species	Working Concentration	Staining Pattern
Annexin II	Santa Cruz	sc-9016	Rabbit Polyclonal Antibody	1:100	membrane and cytoplasm
cofilin 1	PTGlab	10960-1-AP	Rabbit Polyclonal Antibody	1:50	cytoplasm
ezrin	Neomarkers	MS-661	Mouse Monoclonal Antibody	1:100	cytoplasm
fascin	DAKO	M3567	Mouse Monoclonal Antibody	1:100	cytoplasm
kindlin-2	Origene	TA500505	Mouse Monoclonal Antibody	1:50	cytoplasm and nucleus
moesin	Abcam	Ab52490	Rabbit Monoclonal Antibody	1:200	cytoplasm
MTSS1	Abgent	AT2933a	Mouse Monoclonal Antibody	1:1000	cytoplasm and nucleus
myosin-9	Santa Cruz	sc-98978	Rabbit Polyclonal Antibody	1:500	cytoplasm
profilin-1	Abcam	ab124904	Rabbit Monoclonal Antibody	1:1000	cytoplasm and nucleus
Rac1	Cytoskeleton	ARC03	Mouse Monoclonal Antibody	1:20	cytoplasm and nucleus
radixin	Abcam	Ab52495	Rabbit Monoclonal Antibody	1:200	cytoplasm
ROCK2	Santa Cruz	sc -100425	Mouse Monoclonal Antibody	1:500	cytoplasm
talin	Santa Cruz	sc-28542	Mouse Monoclonal Antibody	1:50	cytoplasm
tensin	Santa Cruz	sc-28542	Rabbit Polyclon Antibody	1:50	cytoplasm
villin 1	Proteintech	66096-1-Ig	Mouse Monoclonal Antibody	1:200	cytoplasm

### Prognostic Significance of 15 Biomarkers and Clinicopathological Characteristics

**Figure 2:**
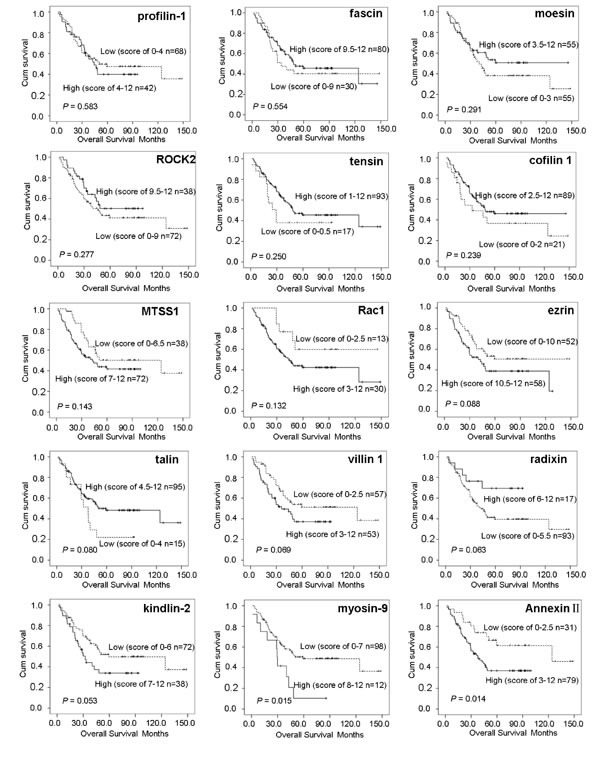
Kaplan-Meier analysis of overall survival for 15 kinds of cytoskeleton proteins in the generation dataset of 110 cases

**Figure 3:**
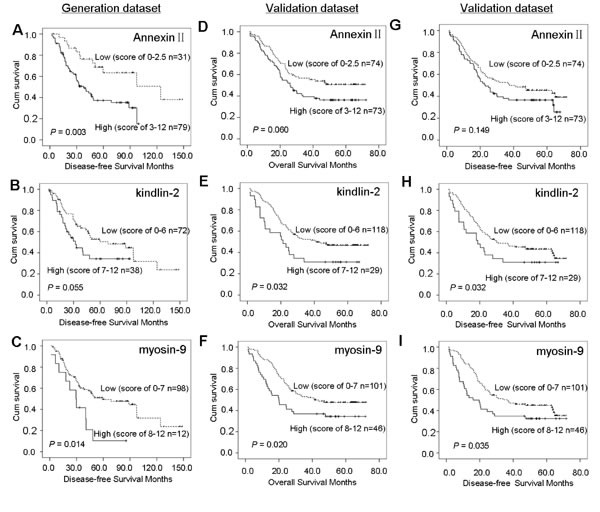
Kaplan-Meier analysis of survival for Annexin II, kindlin-2 and myosin-9 in the two datasets In the generation dataset, disease-free survival is shown for the 110 patients with ESCC (A, B and C). In the validation dataset, overall survival and disease-free survival are shown for the 147 patients with ESCC (D, E, F and G, H, I).

The 1-, 3-, and 5-year OS and DFS percentages, for the generation dataset, were 87.3% and 88.0%, 59.3% and 57.9%, and 44.4% and 45.0%, respectively. According to the Kaplan-Meier analysis, Annexin II and myosin-9 were closely associated with OS among patients with ESCC, and kindlin-2 was of only borderline significance in the relatively small generation cohort. However, the remaining 12 biomarkers had no statistical significance between protein expression and clinical outcome of ESCC (Figure 2). Kaplan-Meier analysis for DFS showed that three biomarkers (Annexin II, kindlin-2 and myosin-9) correlated with recurrence (Figure 3A-3C). On univariate analyses, the three biomarkers and two clinicopathological factors (regional lymph nodes and TNM classification) were all confirmed as prognostic factors for OS and DFS, while other clinicopathological indexes (age, gender, tumor location, tumor size, primary tumor, histologic grade and therapy) had no prognostic significance for OS and DFS (Table 2).

**Table 2:** Univariate and multivariate analyses of factors associated with overall survival (OS) and disease-free survival (DFS) in the generation and validaton datasets

Variables	Generation dataset				Validation dataset			
	OS		DFS		OS		DFS	
	HR (59%CI)	*P*	HR (59%CI)	*P*	HR (59%CI)	*P*	HR (59%CI)	*P*
Univariate analyses								
Age (>58 vs. ≤58)	1.22(0.72 to 2.05)	0.454	1.32(0.80 to 2.21)	0.280	1.09(0.71 to 1.68)	0.699	0.98(0.65 to 1.48)	0.917
Gender (Female vs. Male)	0.83(0.46 to 1.50)	0.543	0.77(0.43 to 1.38)	0.382	0.91(0.55 to 1.52)	0.723	0.95(0.59 to 1.53)	0.827
Tumor Location		0.806		0.702		0.593		0.851
Upper vs. Middle	0.98(0.35 to 2.73)	0.967	0.71(0.22 to 2.29)	0.569	0.58(0.21 to 1.64)	0.308	1.07(0.48 to 2.40)	0.873
Lower vs. Middle	0.79(0.38 to 1.61)	0.512	0.78(0.38 to 1.60)	0.499	0.93(0.59 to 1.45)	0.743	0.90(0.58 to 1.39)	0.636
Tumor Size		0.541		0.354		0.416		0.481
3-5cm vs. ≤3cm	1.37(0.73 to 2.59)	0.329	1.35(0.73 to 2.51)	0.341	1.34(0.77 to 2.33)	0.304	1.26(0.75 to 2.13)	0.378
>5cm vs. ≤3cm	1.54(0.60 to 3.98)	0.373	1.89(0.77 to 4.63)	0.164	1.51(0.81 to 2.81)	0.197	1.43(0.79 to 2.57)	0.233
Primary Tumor (T3+T4 vs. T1+T2)	2.09(0.51 to 8.58)	0.305	1.38(0.43 to 4.43)	0.585	1.07(0.57 to 2.02)	0.835	1.19(0.63 to 2.24)	0.583
Histologic Grade		0.758		0.416		0.033		0.012
G2 vs. G1	0.84(0.47 to 1.48)	0.540	0.77(0.45 to 1.33)	0.346	1.96(0.94 to 4.09)	0.073	2.30(1.11 to 4.79)	0.026
G3 vs. G1	0.71(0.24 to 2.11)	0.542	0.49(0.14 to 1.64)	0.245	3.38(1.36 to 8.41)	0.009	3.91(1.59 to 9.60)	0.003
Regional Lymph Nodes (N1+N2+N3 vs. N0)	2.69(1.56 to 4.63)	0.000	2.52(1.49 to 4.26)	0.001	2.45(1.52 to 3.95)	0.000	2.62(1.66 to 4.14)	0.000
TNM classification (III+IV vs. I+II)	2.79(1.62 to 4.78)	0.000	2.43(1.45 to 4.09)	0.001	2.34(1.48 to 3.70)	0.000	2.54(1.63 to 3.94)	0.000
Therapy (Comprehensive Therapy vs. Only Surgery)	1.28(0.57 to 2.86)	0.549	0.86(0.36 to 2.06)	0.733	0.60(0.36 to 1.01)	0.055	0.80(0.50 to 1.28)	0.350
Annexin II (high vs. low)	2.24(1.16 to 4.35)	0.017	2.62(1.35 to 5.08)	0.005	1.52(0.98 to 2.35)	0.062	1.36(0.89 to 2.05)	0.151
kindlin-2 (high vs. low)	1.69(0.99 to 2.88)	0.056	1.68(0.98 to 2.85)	0.058	1.73(1.04 to 2.86)	0.034	1.51(0.92 to 2.49)	0.103
myosin-9 (high vs. low)	2.29(1.15 to 4.56)	0.018	2.31(1.16 to 4.60)	0.017	1.69(1.08 to 2.65)	0.022	1.59(1.03 to 2.46)	0.036
3-protein signature (high vs. low)	2.74(1.60 to 4.70)	0.000	2.78(1.62 to 4.77)	0.000	1.85(1.20 to 2.86)	0.005	1.65(1.09 to 2.51)	0.018
Multivariate analysis								
Histologic Grade						0.035		0.009
G2 vs. G1					1.74(0.83 to 3.63)	0.142	2.12(1.02 to 4.41)	0.045
G3 vs. G1					3.27(1.31 to 8.15)	0.011	4.08(1.65 to 10.1)	0.002
Regional Lymph Nodes (N1+N2+N3 vs. N0)	2.36(1.36 to 4.11)	0.002	2.22(1.30 to 3.80)	0.003	2.29(1.42 to 3.70)	0.001	2.50(1.58 to 3.96)	0.000
3-protein signature (high vs. low)	2.34(1.34 to 4.05)	0.002	2.41(1.40 to 4.17)	0.002	1.70(1.10 to 2.64)	0.017	1.51(0.99 to 2.29)	0.055

NOTE. Multivariate analysis, Cox proportional hazards regression model. Variables were adopted for their prognostic significance by univariate analysis.

### The Predictive Model Based on the Three Biomarkers

A three-protein signature model, involving Annexin II, kindlin-2, and myosin-9, resulted from our analysis. The risk score of 3-protein signature predictive model was calculated as described in the equation:

Y=(β1) ×(Annexin II)+( β2) ×(Kindlin-2)+( β3) ×(Myosin-9)

Here, Y is the outcome final predictive risk score and βn is its corresponding regression coefficient, using univariate Cox proportional hazards regression analysis. The corresponding coefficients were as fellows: β1=0.808, β2=0.522 and β3=0.829. The 50th percentile (median) of the final risk scores was 0.808 (0 to 2.159). All patients were ranked and divided into high-risk (risk score >0.808) and low-risk groups (risk score ≤ 0.808).

**Figure 4:**
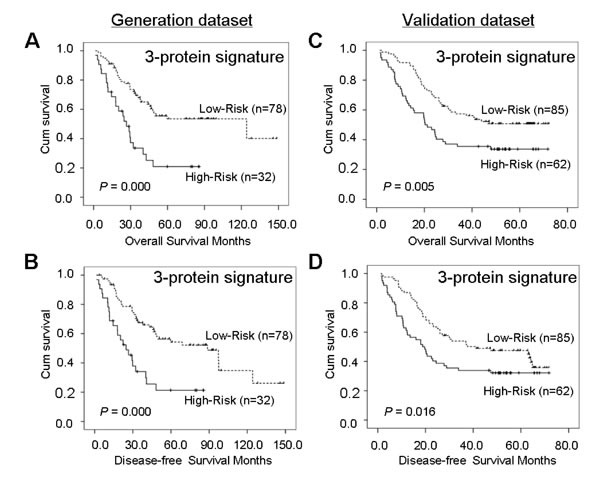
Kaplan-Meier analyses of overall survival and disease-free survival for the three-protein signature model in the generation dataset of 110 cases (A and B) and in the validation dataset of 147 cases (C and D)

The 5-year OS and DFS rates in high-risk group were significantly lower than those in low-risk group (21.0% and 21.3% vs. 53.5% and 54.3%, *P*=0.000 and *P*=0.000, Figure 4A, 4B). Using univariate analyses, we found a significant correlation between the three-protein signature and prognosis in all the cases (*P*=0.000 for OS and DFS, Table 2). According to multivariable Cox proportional hazard regression analysis, both regional lymph nodes and the 3-protein signature were strong and independent prognostic factors for both OS and DFS (for regional lymph nodes, hazard ratio=2.36 [95% CI, 136-4.11], *P*=0.002 and hazard ratio=2.22 [95% CI, 1.30-3.80], *P*=0.003; for the 3-protein signature, hazard ratio=2.34 [95% CI, 1.35-4.05], *P*=0.002 and hazard ratio=2.41 [95% CI, 1.40-4.17], *P*=0.002, Table 2).

### Predictive Power of the Predictive Model

**Figure 5:**
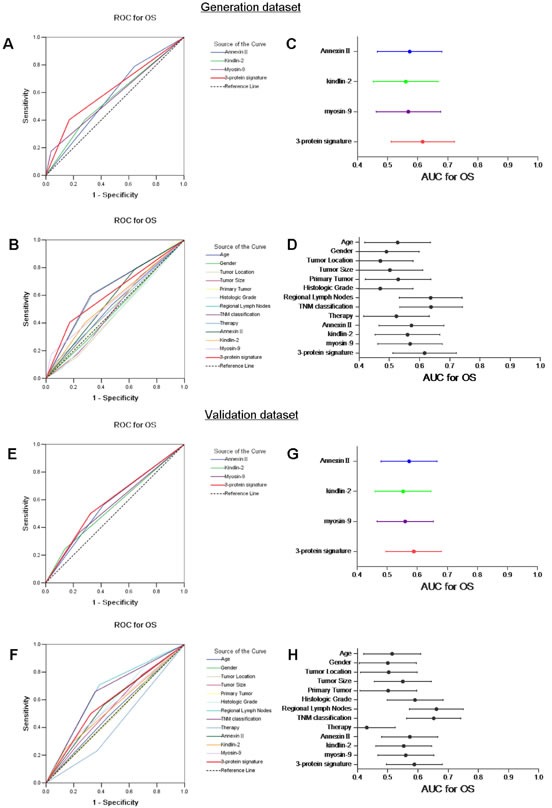
Predictive ability of the 3-protein signature model compared with single biomarkers and other clinical prognostic indices according to receiver operating characteristic (ROC) curves and areas under the curve (AUCs) with 95% CI in the generation dataset (A, B and C, D) and the validation dataset (E, F and G, H)

In comparison with a single biomarker, the predictive power of the 3-protein signature was more robust than that of Annexin II, kindlin-2, or myosin-9 alone, as revealed by ROC curve analysis (Figure 5A, 5C). The specificity and sensitivity were 83% and 40.4%, respectively, and the area under the curve (AUC) with 95% CI for OS was 0.617. Moreover, the predictive power of the 3-protein signature, which almost same as that of regional lymph nodes and TNM classification, was higher than other clinicopathological indices (age, gender, tumor location, tumor size, primary tumor, histologic grade and therapy) (Figure 5B, 5D).

### Validation of the Predictive Model

We validated the 3-protein signature predictive model by TMAs on another independent cohort of 147 specimens with ESCC. The results were similar to those in the generation dataset. Annexin II, kindlin-2, and myosin-9 were optimized successfully to stain tissue and were prognostic in the validation dataset, but borderline significant for Annexin II (Figure 3D-3F). Only kindlin-2 and myosin-9 were closely related to tumor recurrence (Figure 3G-3I). ESCC patients in the high-risk group had a shorter 5-year OS and DFS than those in low-risk group (33.7% and 32.2% vs. 50.8% and 47.7%, *P*=0.005 and *P*=0.016, Figure 4C, 4D). On univariate analyses, the three biomarkers and four clinicopathological factors (histologic grade, regional lymph nodes, TNM classification and therapy) were all confirmed as prognostic factors for OS, while only myosin-9 and three clinicopathological factors (histologic grade, regional lymph nodes and TNM classification) were risk factors for tumour recurrence of ESCC. And using univariate analyses, we also found a significant correlation between the three-protein signature and prognosis in all the cases (*P*=0.005 for OS and *P*=0.018 for DFS). In multivariate analysis, the 3-protein signature model was still a strong and independent predictor of OS and DFS in validation dataset (hazard ratio= 1.70 [95% CI, 1.10-2.64], *P*=0.017 and hazard ratio=1.51 [95% CI, 0.99-2.29], *P*=0.055), together with histologic grade and regional lymph nodes (Table 2). The *P* values for Annexin II indicated some difference between generation dataset and validation dataset, which may be due mainly to the sample size and limited follow-up time. The ROC curve analysis revealed that the predictive ability of the 3-protein signature was higher than that of a single biomarker and other clinicopathological indices, and approximated regional lymph nodes and TNM classification (Figure 5E-5H).

### Combination of the three-protein signature and TNM classification

**Figure 6:**
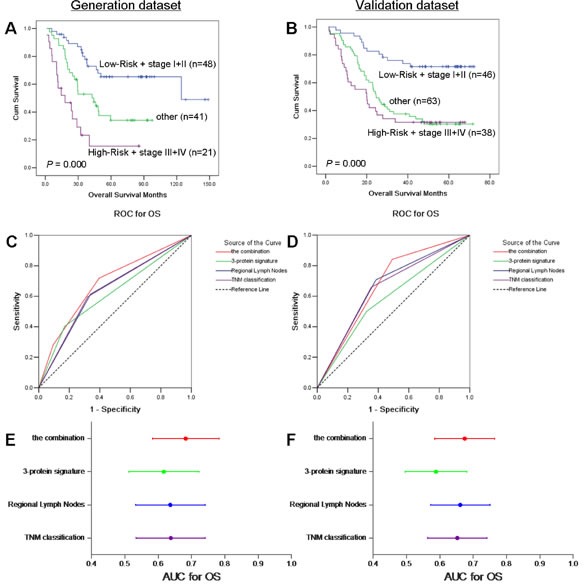
Kaplan-Meier analysis and predictive ability of the combination of the 3-protein signature and TNM classification in the generation and validation datasets Kaplan-Meier analyses of overall survival for the combination of the 3-protein signature and TNM classification in two datasets (A and B). Predictive ability of the combination compared with 3-protein signature, regional lymph nodes and TNM classification by receiver operating characteristic (ROC) curves (C and D) and areas under the curve (AUCs) with 95% CI are shown in E and F.

Our results indicated that there is a significant correlation between the three-protein signature and prognosis in all the cases. In current clinical practice, TNM classification is considered the optimal prognostic indicator. Therefore, we next considered these characteristics together. Patients were subdivided into three subgroups: low-risk + stage I+II, high-risk + stage III+IV, and other (low-risk + stage III+IV and high-risk + stage I+II). Those high-risk + stage III+IV patients had the poorest prognosis, while the low-risk + stage I+II subgroup had the best prognosis. Kaplan-Meier curves showed significant differences in OS among the three groups (*P*=0.000 in generation dataset and validation dataset, Figure 6A, 6B). However, cumulative survival time was to be the same between the high-risk + stage III+IV subgroup and other subgroup at about 50 months in validation dataset. That the median follow-up time of validation dataset was 28.8 months might be the reason. The ROC curve analysis indicated that the combination was most robust among four indices (the combination of the three-protein signature and TNM classification, 3-protein signature, regional lymph nodes and TNM classification) (Figure 6C-6F).

## DISCUSSION

This study generates and internally validates a 3-protein signature predictive model that predicts prognosis and tumor recurrence in two entirely independent cohorts of ESCC patients. The methodology performed in our predictive model is a cross-validation approach based on immunohistochemistry and statistical analysis. The reason for adopting this methodology is that formalin-fixed, paraffin-embedded tissue has far greater availability than other types of samples, such as fresh-frozen samples, and immunohistochemistry is rapid, convenient, robust, technically simple, easy to interpret, reproducible, and cost-effective for clinical practice, unlike gene expression at the mRNA level, for example.

Molecular signatures are reported frequently and have proven to be prognostic in various tumors. Molecular signatures, similar to ours, have been shown to predict survival and/or tumor recurrence in breast cancer [10, 11], lung cancer [12], colorectal cancer [13], hepatocellular carcinoma [14, 15], bladder cancer [16], classic Hodgkin's lymphoma [17], neuroblastoma [18] and even esophageal and gastroesophageal junctional adenocarcinoma [19]. In ESCC, however, the application of molecular signatures is less advanced. Therefore, this study provides a useful framework for future work on generating similar prognostic models.

The biomarkers involved in this study were carefully selected according to our previous studies and other reports. The cytoskeleton, which plays a prominent role in the cell cycle, morphogenesis and migration, is closely associated with human tumors [20]. Recent studies from our laboratory and others have identified more than 200 different cytoskeleton constituents or binding proteins. However, only about 45 kinds have been reported in ESCC [21]. Hence, the 15 biomarkers used here include biomarkers that are reported for the first time. In addition, our 15 biomarkers all belong to actin-binding proteins [22]. Annexin II is a member of the calcium-dependent phospholipid-binding protein family, plays a role in the regulation of cellular growth and in signal transduction pathways, and is overexpressed in hepatocellular carcinoma [23], colorectal cancer [23, 24], breast cancer [23, 25] and ESCC [23, 26], and has also been identified as a potential target for therapy [27, 28]. Our result showed the same as Zhang X and Feng JG [23, 26]. Kindlin-2 is a member of the kindlin family of focal adhesion proteins, and is involved in integrin signaling and linkage of the actin cytoskeleton to the extracellular matrix. Although few studies report on kindlin-2, it has been found to be up-regulated in breast and gastric cancer cell lines [29, 30], but down-regulated in leiomyosarcomas [31] and mesenchymal cancer cells [32]. Targeting kindlin-2 may improve drug efficacy and reduce the dose of drug required to treat prostate cancer [33]. Our results reveal that over-expression of kindlin-2 is associated with poor prognosis of ESCC. Myosin-9 is a myosin IIA heavy chain, and is involved in several important functions, including cytokinesis, cell motility and maintenance of cell shape. Myosin-9 has been reported to be a target for anti-invasive treatment in human MCF-7 breast cancer cells [34] and gastric cancer [35]. High expression of myosin-9 is correlated with short survival in lung adenocarcinoma and ESCC [36], similar to our results.

The 3 biomarkers are heterogeneously expressed in clinical samples, and the positive frequency of a single biomarker is commonly <10%. No patient expressed all the 15 biomarkers and only 7.0% (18/257) patients expressed the three biomarkers simultaneously. Therefore, it is reasonable to combine multiple biomarkers instead of an individual marker, which will significantly increase both the predictive range and power of the predictive model.

In this study, the three markers (Annexin II, kindlin-2 and myosin-9) combined prove to be significant predictors of OS and DFS. ESCC patients predicted to be high-risk had a very poor prognosis and were more prone to experience tumour recurrence. The predictive power of the 3-protein signature closely approached the predictive power of clinical staging, and that of the combination of the 3-protein signature and TNM classification was stronger than clinical staging. The 3 biomarkers employed in our predictive model may provide a novel therapeutic candidate for ESCC. Certainly, this study is a retrospective study that is limited to patients with ESCC undergoing curative resection. Prospective studies involving larger populations will be required to further validate the usefulness of this system. In conclusion, we demonstrate a clinically applicable prognostic model that accurately predicts ESCC patient survival and/or tumor recurrence, and thus can serve as a complement to current clinical risk stratification approaches.

## MATERIALS AND METHODS

### Patients and Tissue Specimens

Two independent datasets (ESCC tissues) were randomly collected from patients with ESCC undergoing curative resection at Shantou Central Hospital from 2000 to 2006 (generation dataset, n=110) and from 2007 to 2009 (validation dataset, n=147), and embedded in paraffin. Patients in the generation dataset were followed up for a maximum period of 148.7 months and a median of 36.5 months, while follow-up of patients in the validation dataset was terminated on 20 November 2013 at a median of 28.8 months. Overall survival (OS) was defined as the interval between surgery and death or the last observation taken. Disease-free survival (DFS) was defined as the interval between the date of surgery and the date of diagnosis of any type of relapse or death. All the tumors were confirmed as ESCC by the pathologists in the Clinical Pathology Department of the Hospital, and the cases were classified according to the seventh edition of the tumor-node-metastasis (TNM) classification of the International Union against Cancer. Evaluation of tumor differentiation was based on histological criteria of the guidelines of the WHO Pathological Classification of Tumors. Information on age, gender, stage of disease, therapy and histopathological factors (such as tumor location, tumor size, primary tumor, histologic grade, regional lymph nodes) was obtained from the medical records. Patient data is summarized in Table 3, and there is no statistical difference between the two datasets using the chi-squared test. The study was approved by the ethical committee of the Central Hospital of Shantou City and the ethical committee of the Medical College of Shantou University, and written informed consent was obtained from all surgical patients to use resected samples for research.

**Table 3:** Clinicopathological characteristics of the generation and validation datasets of patients with ESCC

Clinical and pathological indices	Generation dataset		Validation dataset		Test statistic	df	*P*
	No.	%	No.	%			
Specimens	110		147				
Median follow-up (Months)	36.5		28.8		T=22.77		0.000^a^
Age (year)							
≤58	55	50	79	53.7	χ2=0.353	1	0.552
>58	55	50	68	46.3			
Gender							
Male	80	72.7	113	76.9	χ2=0.578	1	0.447
Female	30	27.3	34	23.1			
Tumor Location							
Upper	8	7.3	10	6.8	χ2=31.83	2	0.000
Middle	81	73.6	59	40.1			
Lower	21	19.1	78	53.1			
Tumor Size							
m≤3cm	32	29.1	38	25.8	χ2=31.64	3	0.000
3-5cm	45	40.9	71	48.3			
>5cm	11	10	36	24.5			
unknown	22	20	2	1.4			
Primary Tumor (T)							
T1+T2	7	6.4	20	13.6	χ2=3.510	1	0.061
T3+T4	103	93.6	127	86.4			
Histologic Grade (G)							
G1	33	30	23	15.6	χ2=7.640	2	0.022
G2	67	60.9	109	74.2			
G3	10	9.1	15	10.2			
Regional Lymph Nodes (N)							
N0	57	51.8	64	43.5	χ2=1.732	1	0.188
N1+N2+N3	53	48.2	83	56.5			
TNM classification							
IA+IB+IIA+IIB	59	53.6	70	47.6	χ2=0.911	1	0.340
IIIA+IIIB+IIIC+IV	51	46.4	77	52.4			
Therapy							
Only Surgery	99	90	104	70.7	χ2=14.05	1	0.000
Comprehensive Therapy^b^	11	10	43	29.3			

NOTE. Comparisons between groups were performed using the chi-squared test unless otherwise indicated.

a One-Sample T Test.

b Comprehensive therapy including surgery + chemotherapy, surgery + radiotherapy and surgery + chemotherapy + radiotherapy.

### Tissue Microarrays (TMAs) and Immunohistochemistry

Markers that were used included Annexin II, cofilin 1, ezrin, fascin, kindlin-2, moesin, Metastasis suppressor 1 (MTSS1), myosin-9, profilin-1, Ras-related C3 botulinum toxin substrate 1 (Rac1), radixin, Rho-associated coiled-coil containing protein kinase 2 (ROCK2), talin, tensin and villin 1. These markers were analyzed in a test cohort of 110 formalin-fixed, paraffin-embedded esophageal curative resection specimens (generation dataset) using tissue microarrays (TMAs), and then, Annexin II, kindlin-2 and myosin-9, which were significantly related to clinical outcome in TMA analysis of the generation dataset, were further validated by the validation dataset TMAs.

TMA construction has been described in our previous studies [37-40]. Two tissue cores of 1.8 mm in diameter were taken from the donor blocks and transferred to the recipient paraffin block at defined array positions. These microarrays were serially sectioned (4 μm) and stained with hematoxylin and eosin to ensure tissue sampling and completeness. The unstained sections were baked overnight at 56°C in preparation for immunohistochemistry staining.

Primary antibodies used in this study are shown in Table 1. Immunohistochemistry was carried out by a two-step protocol (PV-9000 Polymer Detection System, ZSGB-BIO, Beijing, China) as previously described [40].

### Evaluation of Immunohistochemical Variables

Tissue sections were independently and blindly assessed by three independent histopathologists (Cao HH, Wang SH, and Shen JH). Discrepancies were resolved by consensus. Positive reactions were defined as those showing brown signals in the cell cytoplasm. Each separate tissue core was scored on the basis of the intensity and area of positive staining. The intensity of positive staining was scored as follows: 0, negative; 1, weak staining; 2, moderate staining; 3, strong staining. The rate of positive cells was scored on a 0–4 scale as follows: 0, 0–5%; 1, 6–25%; 2, 26–50%; 3, 51–75%; 4, >75%. If the positive staining was homogeneous, a final score was achieved by multiplication of the two scores, producing a total range of 0–12. When the staining was heterogeneous, we scored it as follows: each component was scored independently and summed for the results. For example, a specimen containing 25% tumor cells with moderate intensity (1×2=2), 25% tumor cells with weak intensity (1×1=1), and 50% tumor cells without immunoreactivity (2×0=0), received a final score of 2+1+0=3. The mean value of the two scores was considered representative of one tumour. For statistical analysis, we had each kind of protein expression score grouped into two subgroups, high-expression and low-expression, according to X-tile [41].

### Construction of a Weighted OS Predictive Score Algorithm

We had used a univariate Cox proportional hazards regression analysis to evaluate the association between clinical outcome and the expression of each biomarker. Subsequently, we developed a model for estimation of prognosis risk similar to what was described as follows. A patient's poor prognosis risk score was then derived by the summation of each biomarker's expression level (high-expression=1, low-expression=0) multiplied by its corresponding regression coefficient [42-44]. All patients were then dichotomized into high-risk and low-risk groups using the 50th percentile (median) cut-off of the final risk score as the threshold value.

### Statistical Analysis

Statistical analyses were performed using SPSS 13.0 for Windows. Comparisons between groups were performed using the chi-squared test and one-sample t test. Cumulative survival time was calculated by the Kaplan-Meier method and analyzed by the log-rank test. Univariate and multivariate analyses were based on the Cox proportional hazards regression model. Receiver operating characteristic (ROC) curve analysis was used to determine the predictive value of the parameters, and the differences in the area under the curve (AUC) were detected by using GraphPad Prism 5. The Kendall tau-b rank correlation analysis was used to evaluate the association between our 3-gene signature expression and clinicopathological factors. A *P* value of less than 0.05 was considered statistically significant.
